# Dr. Joseph Eastman

**Published:** 1902-07

**Authors:** 


Dr. Joseph Eastman, of Indianapolis, died June 5, 1902, aged 60 years. He was born in Fulton County, N.Y., and enlisted in the 77th New York volunteer infantry, early in the civil war. Later he served as assistant surgeon and after being mustered out of service he located in Indiana. He became an abdominal surgeon of prominence and was dean of one of the medical colleges at Indianapolis. Among his surviving kin are two sons, Thomas Barker and J. Rilus Eastman, who are physicians of rising fame in that city.

